# Eucalyptus

**Published:** 1882-09

**Authors:** 


### SELECTIONS.

### EUCALYPTUS.

The following, from a paper read before the Massachusetts Dental Society, June 8, 1882, is by Dr. Geo. L. Parmele, M.D.D., M.D., emphasises with considerable force the value of eucalyptus. The suggestions in regard to the proper mode of use, and the cases to which it is applicable are worthy of special attention:

"The preparations of eucalyptus in use are *tinctura eucalypti extractum eucalypti* and *eucalyptol*, which latter is the essential oil of the leaves, and the physiological actions of eucalyptus are due to this oil. It has a warm, aromatic taste, and increases the flow of the saliva. Among the diseases in which it is said to be used with benefit are pulmonary gangrene, hay fever, palpitations frequent in women about the change in life, hysteria, etc. But the most important use (internally) is in treatment of catarrhal affections of the mucous membranes; especially catarrh of the bladder.

As an antiseptic of high order in the treatment of foul ulcers, abscesses and sloughing wounds, the superiority of eucalyptol has already been proven both in this country and in Europe. It is a much more powerful antiseptic than carbolic acid; its effects more permanent; it has no irritating or caustic action, and thus possesses all the advantages of an antiseptic, with none of its unfavorable properties.

While a few object to its taste, I find that nearly all find its taste far less objectionable than carbolic acid. Mr. David Hepburn, in a paper entitled, "Chronic Suppuration Connected with the Teeth," read before the Odontological Society of Great Britain, gives great praise to eucalyptol for its marvelous power as an antiseptic agent. In a case of abscess, where there had been an incessant discharge of putrid pus in large quantities, he found that eucalyptus rendered everything pure as nothing else could do, and whenever its use was discontinued there was a return of the discharge. Dr. Rollins, of Boston, suggests its use in a mouth wash, as follows:

℞. Sodæ boratis, 15 grams;

Olei eucalypti, 2 grams;

Magnesiæ carbonatis, 4 grams;

Aquæ, 1,000 grams.

M.

A. S. Underwood, M.R.C.S.,L.D.S., England, in the *Monthly Review of Dental Surgery* (London), recommends it very highly in treatment of dead roots, and abscesses about the mouth, using it alone or in combination with iodoform. I have used iodoform with it, but thinking I succeed as well with eucalyptus alone, and as the smell of iodoform is so disagreeable and sickening, I have abandoned this combination. For the benefit, however, of those who desire to try it, I give the following formula, taken from the *Louisville Medical News*, which it claims will effectually mask the odor of iodoform:

℞. Iodoform, ʒss;

Oil lavender, gtt x;

Alcohol, flʒii;

Glycerine, flʒvi.

M.

I have used eucalyptus now for two years, and the results obtained in the treatment of dead pulps, alveolar abscesses, and diseased antra, warrant me in highly recommending it to my brother practitioners. I use an extract of the leaves prepared by Sander and Son, Sandhurst, Australia, they being, I think, the sole and exclusive manufacturers. It should not be confounded with *ol euc. c. ligno* (wood oil). It can be had of reliable druggists in any large city.

It is my custom, after a pulp has been *thoroughly* devitalized, to carefully adjust the rubber dam and so prepare the crown that free and as direct access as possible be obtained to the pulp cavity and canals; then, by means of the usual nerve instruments, I carefully enlarge the canals and remove all that is possible of the devitalized pulp. Having accomplished this, I repeatedly wash out the canals with alcohol carried up by means of cotton on fine broaches, and pumped up also. Then, by means of fine needles of bibulous paper and the hot-air syringe, I thoroughly dry the cavity and canals. My next step is to force eucalyptus up in the same manner as the alcohol. If there has been no periodontitis, I at once proceed to introduce gutta-percha into the roots. This I accomplish by warming small bits of gutta-percha and, with the aid of a spatula, rolling them on a porcelain slab until they are reduced to fine wires, pointed at one end. These gutta-percha wires I dip, while warm, in eucalyptus, and immediately insert them into the canals, forcing them up to place with fine, smooth broaches. It will be found that enough gutta-percha will be dissolved to form a paste-like mass, which will be forced up into the minutest parts of the canal. This part of the operation should be gently performed, so as to avoid forcing beyond the apex. In cases where there has been previous trouble, I pack the roots with cotton and eucalyptus, and insert a trial filling, for a few days, of gutta-percha. After packing the canals, I generally dry out, and fill the remainder of the cavity with oxyphosphate cement until such time as I am prepared to insert the permanent filling in the crown. Always, after finishing the root fillings, I paint gums with iodine. It was formerly my custom to cleanse the roots slowly, at different sittings, but I think I have been more successful since I adopted the immediate and thorough method. My records show that in the past two years I have developed no after inflammation, which was not easily arrested. Where constitutional treatment is required to arrest periodontitis, I obtain the best results from aconite and belladonna.

In reply to D.D.S.'s second question, I will simply say that I consider the *permanent* filling of root canals with cotton, and any medical preparation, a pernicious custom, although I have seen cases where it has been successfully accomplished for years. In the treatment of abscesses, I generally use eucalyptus in the same manner as carbolic acid, or any other antiseptic. I find that a very convenient way to dilate the fistulous opening of an abscess is by using suitable sizes of violin strings, dipped into eucalyptus, and immediately introduced into the fistula as far as possible, cutting off even with the opening of the fisula. The next day it will be found swollen, and the fistula dilated. If necessary, a larger size can then be introduced until the desired dilation is accomplished. Small medicated bougies introduced into these fistulous openings are often a convenient method of medication."

